# Dupilumab treatment is not associated with changes in lymphoma risk in atopic dermatitis and other type 2 inflammatory diseases: data from a large-scale retrospective cohort study

**DOI:** 10.3389/fmed.2025.1702736

**Published:** 2026-01-20

**Authors:** Khalaf Kridin, Katja Bieber, Henning Olbrich, Dagmar von Bubnoff, Gema Hernandez, Henner Zirpel, Nikolas von Bubnoff, Diamant Thaçi, Ralf J. Ludwig

**Affiliations:** 1 Lübeck Institute of Experimental Dermatology, University of Lübeck, Lübeck, Germany; 2 Unit of Dermatology and Skin Research Laboratory, Galilee Medical Center, Nahariya, Israel; 3 Azrieli Faculty of Medicine, Bar-Ilan University, Safed, Israel; 4 Department of Dermatology, University Clinic Schleswig-Holstein (UKSH), Lübeck, Germany; 5 TriNetX, LLC, Cambridge, MA, United States; 6 Biomedical Informatics Group, Artificial Intelligence Department, E.T.S.I. Informáticos, Universidad Politécnica de Madrid, Madrid, Spain; 7 Institute and Comprehensive Centre for Inflammation Medicine, University-Hospital Schleswig-Holstein, Lübeck, Germany; 8 Department of Hematology and Oncology, University Medical Center Schleswig-Holstein and University Cancer Center Schleswig-Holstein (UCCSH), University of Lübeck, Lübeck, Germany

**Keywords:** atopic dermatitis, dupilumab, lymphoma, mycosis fungoides, real-world, Sézary disease, TriNetX, type 2 inflammatory diseases

## Abstract

**Background:**

The association between atopic dermatitis (AD) and lymphoma risk remains inconclusive. Dupilumab, approved for moderate-to-severe AD, has been linked to an increased lymphoma risk, raising significant concerns.

**Objectives:**

The objective of the study was to clarify the association between AD and lymphoma risk and extend to non-dermatological type 2 inflammatory diseases (T2IDs). This study also aimed to assess the impact of dupilumab on lymphoma risk in AD and non-dermatological T2IDs.

**Methods:**

A retrospective cohort study was conducted using the TriNetX database. Propensity-score matching allowed for better comparability, and sensitivity analyses ensured robustness.

**Results:**

Among 801,508 cases and controls, AD was associated with an increased risk of lymphoma, e.g., cutaneous T-cell lymphoma (CTCL) and non-Hodgkin lymphoma (NHL). Among 14.4 million cases and controls, non-dermatological T2IDs also conferred an increased lymphoma risk. In the comparison of AD patients treated with dupilumab versus other systemic treatments (*n* = 7,840 per group), dupilumab exposure did not alter the risk for lymphomas but tended toward reduced risks. This decreased risk association was most evident in non-dermatological T2IDs (*n* = 16,908 per group).

**Limitations:**

Retrospective data analysis, data quality, possible false registration of ICD-10-codes.

**Conclusion:**

T2IDs, including AD, are associated with a significantly increased risk for lymphoma. Treatment with dupilumab partially ameliorates this risk association, especially for NHL.

## Introduction

Atopic dermatitis (AD) is a chronic inflammatory skin disease characterized predominantly by a type 2 immune response (1–3). In addition to skin inflammation, often accompanied by severe itching, patients with AD bear a substantial comorbidity burden. These comorbidities can be divided into those associated with the “atopic march,” including food allergies, allergic rhinitis, and asthma, and those associated with non-atopic comorbidities (1), including anxiety, autoimmune diseases, infections, malignancies, metabolic syndrome, and cardiovascular diseases (4, 5). Finally, AD has been associated with a slightly but significantly increased risk of all-cause mortality (6).

Lymphomas are another potential comorbidity associated with AD. The first report suggesting an increased lymphoma risk in AD was published in 2005. However, this study did not control for potential confounding factors and did not correct for multiple testing (7). Since then, several studies have demonstrated an elevated risk of lymphoma in AD, while others have failed to confirm this. Studies reporting an increased lymphoma risk also noted a correlation between disease severity and lymphoma risk, with more severe AD linked to a higher likelihood of lymphoma (8–11). A recent nested case–control study (11), matched for age, ethnicity, sex, income, health insurance, disability, education, and smoking, found that AD patients had significantly higher odds of developing non-CTCL. In conclusion, the majority of studies suggest an increased risk of lymphoma in patients with AD. However, a comprehensive systematic analysis that includes both CTCL and non-CTCL lymphomas is lacking. Insights into lymphoma risk in non-dermatological T2IDs are limited: asthma has generally been associated with a reduced risk for non-Hodgkin lymphoma (NHL) (12), whereas COPD has been identified as a risk factor for the development of mycosis fungoides (MF) (13). No data have been published on the lymphoma risk in patients with chronic sinusitis or eosinophilic esophagitis.

Independently, dupilumab has been approved for the treatment of moderate-to-severe atopic dermatitis (14) and other T2IDs (15–18). Recent reports raised concerns that dupilumab may be associated with an increased lymphoma risk, especially in AD: A retrospective cross-sectional single-center study identified five patients who developed MF while receiving dupilumab treatment for AD. Subsequently, a systematic literature review identified 20 more AD patients in whom MF developed during dupilumab therapy (19). In support of this notion, a recent study documented increased odds of CTCL and NHL in AD patients treated with dupilumab compared to those not exposed to the drug (20). While this study garnered significant interest, it also sparked controversy. The primary criticism was that AD patients requiring systemic treatment (such as dupilumab) were compared to all other AD patients not exposed to dupilumab. This has been addressed in one of the response letters to the study by contrasting CTCL risk in dupilumab versus oral prednisone, cyclosporine, or methotrexate-exposed AD patients. In this study, analysis of AD patients on treatments generally used for moderate-to-severe disease did not detect a difference regarding increased CTCL between the two groups (21). Another source of bias in reports based on dermatological T2IDs, especially AD, may be initial misdiagnosis of CTCL as AD, with falsely recorded emergence of CTCL following an AD diagnosis.

To comprehensively assess the lymphoma risk in AD and non-dermatological T2IDs and to clarify the potential risk imposed by dupilumab in lymphoma development, retrospective cohort studies were conducted here.

## Methods

### Study design and database

**Figure 1:**
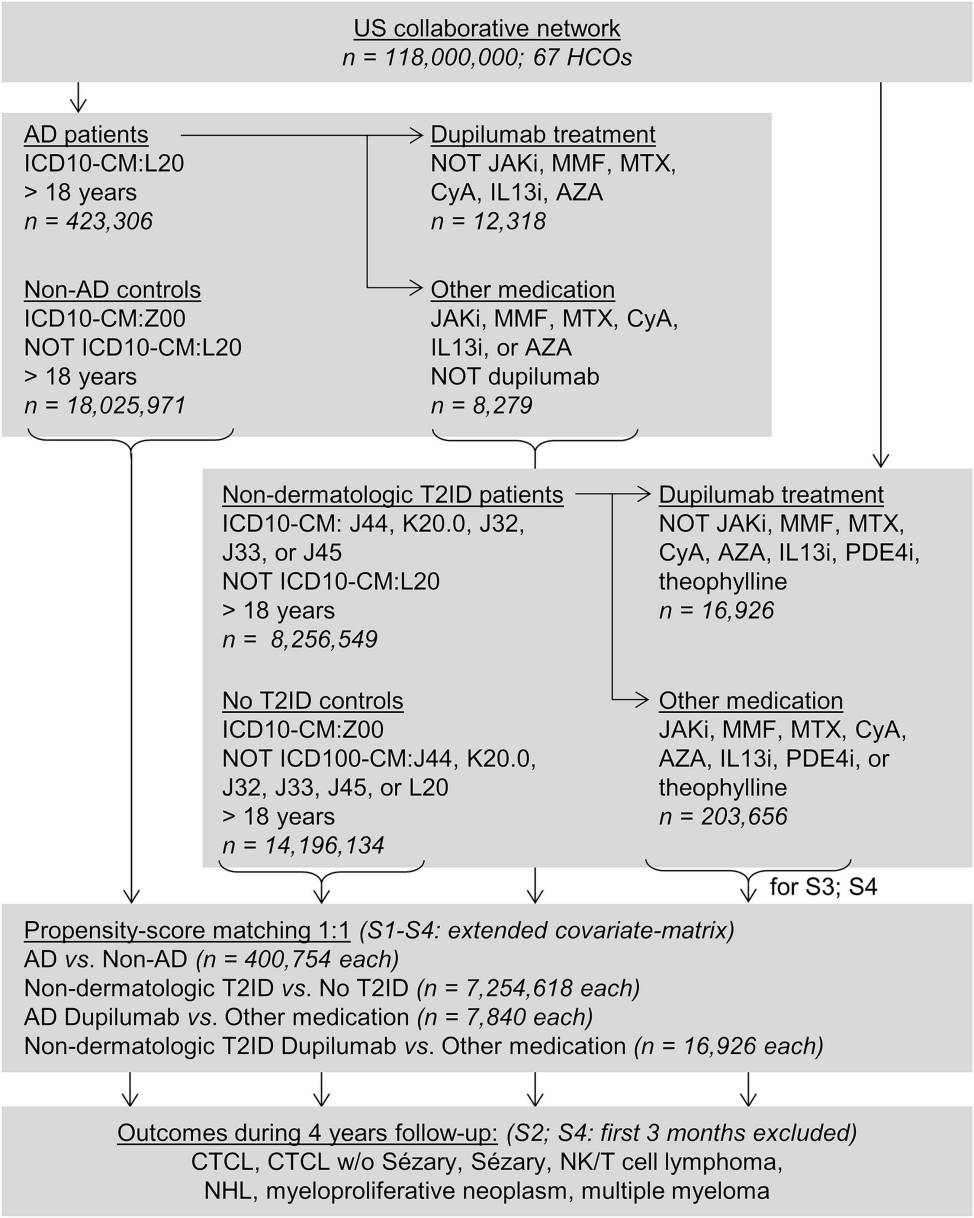
Study inclusion flowchart.

A propensity score-matched retrospective cohort study was conducted (Figure 1) using the US Collaborative Network from the federated electronic health record (EHR) database TriNetX, following previously published protocols (22–24). This network was chosen due to its large volume of documented EHRs and the comprehensive covariate data available (25). Researchers at UKSH have access to the TriNetX database through a collaboration with TriNetX. The US Collaborative Network of TriNetX was used to identify EHRs from patients diagnosed with AD (or non-dermatological T2IDs) and a matched non-AD (non-dermatological T2ID) control group. The risks for CTCL, CTCL excluding Sézary disease, Sézary disease, T/NK cell lymphoma, NHL, myeloproliferative neoplasms, and multiple myeloma were compared between AD patients and non-AD controls using PSM to enhance comparability. Two sensitivity analyses were performed to assess the robustness. To evaluate the potential lymphoma risk associated with dupilumab, these risks were also compared between AD patients who were treated with dupilumab, non-dermatology T2ID patients who were treated with dupilumab, and corresponding groups who received systemic treatments other than dupilumab. Consequently, sensitivity analyses were performed to assess robustness. Endpoints were defined using ICD-10 codes before data collection.

### Ethics statement

The data reviewed is a secondary analysis of existing data, does not involve intervention or interaction with human subjects, and is de-identified per the de-identification standard defined in Section §164.514(a) of the Health Insurance Portability and Accountability Act (HIPAA) Privacy Rule. The process by which the data are de-identified is attested to through a formal determination by a qualified expert as defined in Section §164.514(b)(1) of the HIPAA Privacy Rule. Thus, our study did not require Institutional Review Board approval.

### Use of artificial intelligence

ChatGPT-4o (OpenAI LCC, San Francisco, California, USA) was used to assist in extracting data from tables and revising sections of the manuscript. However, all extracted data and revisions were thoroughly reviewed and validated by the authors. The authors took full responsibility for the accuracy, integrity, and final content of the manuscript.

More detailed information on the methods is provided in Supplementary file 1.

## Results

### Baseline characteristics

In the primary analysis focusing on the lymphoma risk in AD, 423,306 cases and 18,025,971 non-AD controls were retrieved (Supplementary Table 1). For the analysis comparing lymphoma risk between non-dermatological T2IDs and non-T2ID controls, cohort characteristics are shown in Supplementary Table 2. Cohort descriptions in analyses focusing on the impact of dupilumab on lymphoma risk are shown in Table 1.

**Table 1:** Characteristics of cohorts investigating the risks of indicated lymphomas between dupilumab and other systemic treatment-exposed patient cohorts.

Characteristic		Before matching			After matching		
	Definition	Dupilumab	Other systemic treatment	Std. diff.	Dupilumab	Other systemic treatment	Std. diff.
		(Cases)	(Controls)		(Cases)	(Controls)	
Primary analysis							
Number of participants	–	12.318	8.279	–	7.840	7.840	–
Follow-up (days) median (interquartile range)	–	495 (852)	759 (1,146)		493 (839)	756 (1,147)	
Age at index/years (SD)	–	45.9 ± 19.3	53.5 ± 17.9	0.4072	52.8 ± 18.1	52.4 ± 17.7	0.0216
Female (%)	–	55.943	66.3	0.2137	62.666	64.503	0.0382
White (%)	–	53.499	58.22	0.0952	58.776	57.36	0.0287
Sensitivity analyses S1 and S2							
Number of participants	–	13.350	8.959	–	7.956	7.956	–
Follow-up (days) median (interquartile range)	–	496 (851)	756 (1,138)	–	497 (834)	753 (1,145)	–
Age at index/years (SD)	–	45.9 ± 19.3	53.5 ± 17.9	0.4112	52.9 ± 18.4	52 ± 17.8	0.0521
Female (%)	–	56.26	66.45	0.2103	64.13	64.64	0.0108
Male (%)		40.11	30.20	0.2086	33.23	31.91	0.0282
White (%)	–	54.25	58.79	0.0917	59.05	57.62	0.0291
Black or African-American (%)		18.62	16.52	0.0553	17.81	16.81	0.0266
Hispanic or Latino (%)		5.78	7.10	0.0536	4.97	7.08	0.0889
Problems related to housing and economic circumstances (%)	ICD10:Z59	3.52	5.24	0.0839	4.29	4.58	0.0140
Problems related to employment and unemployment (%)	ICD10:Z56	1.27	2.82	0.1103	1.91	2.12	0.0152
Personal history of nicotine dependence (%)	ICD10:Z87.891	11.61	20.26	0.2380	16.78	17.51	0.0193
Nicotine dependence (%)	ICD10:F17	12.10	16.12	0.1157	14.67	15.02	0.0099
Alcohol-related disorder (%)	ICD10:F10	5.59	7.28	0.0689	6.32	6.75	0.0173
Chronic kidney disease (CKD) (%)	ICD10:N18	6.17	15.92	0.3147	10.19	12.37	0.0688
Chronic lower respiratory diseases (%)	ICD10:J40–J4A	31.91	33.54	0.0348	31.37	32.68	0.0280
Body mass index (kg/m^2^)	TNX:9083	29.5 ± 7.47	29.2 ± 7.47	0.0284	29.2 ± 7.23	29.4 ± 7.58	0.0333
Glucocorticosteroids (%)	VA:HS051	81.344	87.772	0.1786	86.899	86.552	0.0103
Sensitivity analyses S3 and S4							
Number of participants	–	16,926	203,656	–	16,926	16,926	–
Follow-up (days) median (interquartile range)	–	488 (790)	880 (1,131)	–	488 (790)	922 (1,106)	–
Age at index/years (SD)	–	51.2 ± 16.4	60.2 ± 14.8	0.5757	51.2 ± 16.4	51.3 ± 16.4	0.0029
Female (%)	–	56.582	64.843	0.1698	56.582	56.723	0.0029
Male (%)	–	40.228	31.751	0.1773	40.228	39.336	0.0182
White (%)	–	69.727	71.244	0.0333	69.727	69.432	0.0064
Black or African-American (%)	–	14.008	13.526	0.014	14.008	13.488	0.0151
Hispanic or Latino (%)	–	5.116	5.874	0.0332	5.116	6.676	0.0663
Problems related to housing and economic circumstances (%)	ICD10:Z59	3.456	4.023	0.0299	3.456	2.765	0.0398
Problems related to employment and unemployment (%)	ICD10:Z56	1.382	1.742	0.029	1.382	0.798	0.0564
Personal history of nicotine dependence (%)	ICD10:Z87.891	18.528	28.183	0.2297	18.528	18.321	0.0053
Nicotine dependence (%)	ICD10:F17	13.258	21.648	0.2224	13.258	12.897	0.0107
Alcohol-related disorder (%)	ICD10:F10	5.43	7.772	0.0944	5.43	4.679	0.0343
Chronic kidney disease (CKD) (%)	ICD10:N18	7.338	22.802	0.4427	7.338	7.415	0.0029
Chronic lower respiratory diseases (%)	ICD10:J40–J4A	77.992	73.924	0.0953	77.992	78.105	0.0053
Body mass index (kg/m^2^)	TNX:9083	31 ± 7.94	30 ± 7.98	0.1232	31 ± 7.94	30.6 ± 8.38	0.0573
Glucocorticosteroids (%)	VA:HS051	84.048	78.513	0.1423	84.048	84.172	0.0034

SD, standard deviation; std. diff., standardized difference.

Characteristics in light gray were not separately included for propensity score matching. Data were retrieved from the US Collaborative Network of TriNetX on 10 July 2024.

### Atopic dermatitis increases the risk of CTCL, Sézary disease, non-Hodgkin lymphoma, and myeloproliferative neoplasms

**Figure 2:**
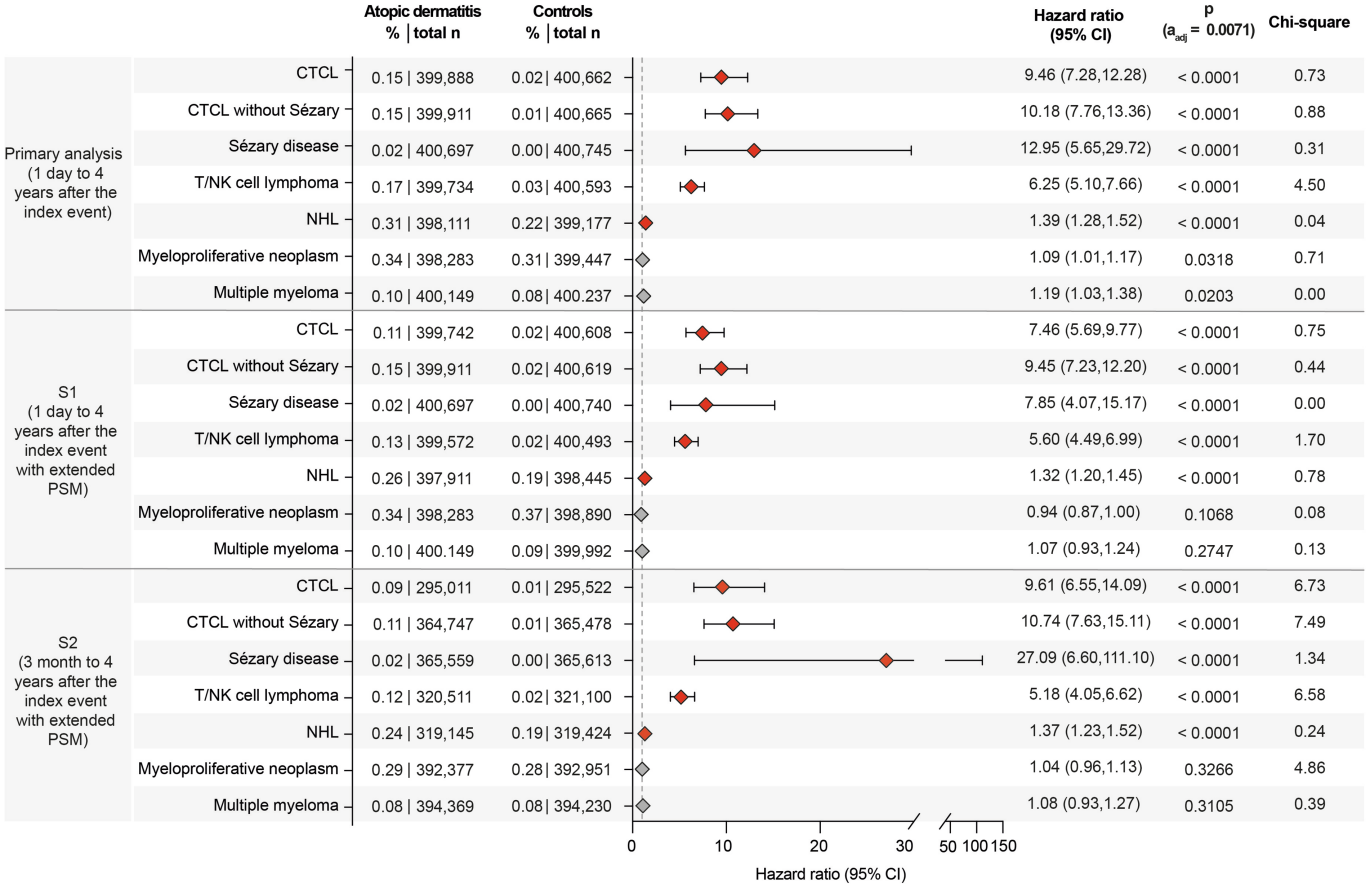
Forest plot of lymphoma risks in patients with atopic dermatitis (AD) and non-AD controls, contrasted across the primary analysis and sensitivity analyses (S1 and S2). The forest plots display the hazard ratios (HR) with 95% confidence intervals (CI) for the risk of the indicated lymphomas. Increased risks were observed for the majority of lymphomas in AD patients, particularly for CTCL and NHL. For myeloproliferative neoplasms and multiple myeloma, risks were elevated but not statistically significant. The boxes correspond to the HR, while error bars represent the 95% CI. Confidence intervals are shown at the 95% level for interpretability. However, statistical significance was assessed using a Bonferroni-adjusted threshold of *α* = 0.0071, and readers are advised to interpret confidence intervals in conjunction with adjusted *p*-values.

In the primary analysis, encompassing over 0.8 million cases and controls, CTCL risk was higher in AD, with 0.147% affected compared to 0.015% in the non-AD control group (HR 9.46, CI 7.28–12.28, *p* < 0.0001). Increased risks in the AD group were also observed for CTCL without Sézary (HR 10.18, CI 7.76–13.36, *p* < 0.0001), Sézary disease (HR 12.95, CI 5.65–29.72, *p* < 0.0001), T/NK cell lymphoma (HR 6.25, CI 5.10–7.66, *p* < 0.0001), and NHL (HR 1.39, CI 1.28–1.52, *p* < 0.0001). These elevated risks persisted across both sensitivity analyses (Figure 2).

### Non-dermatological T2IDs are associated with an increased risk of CTCL, Sézary disease, non-Hodgkin lymphoma, and myeloproliferative neoplasms

**Figure 3:**
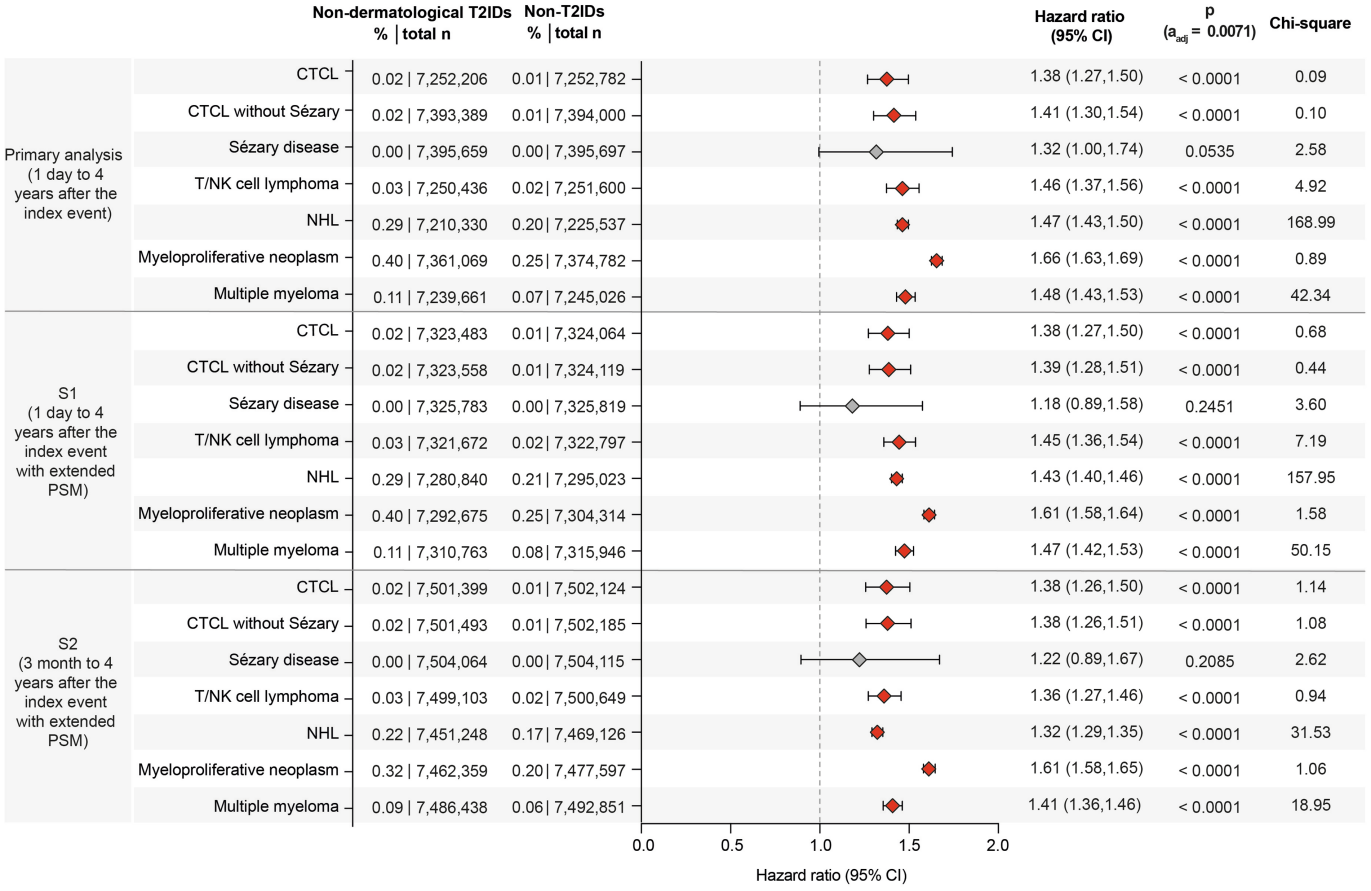
Forest plot of lymphoma risks in patients with non-dermatological type 2 inflammatory diseases (T2IDs) and non-T2ID controls, contrasted across the primary analysis and sensitivity analyses (S1 and S2). The forest plots display the hazard ratios (HR) with 95% confidence intervals (CI) for the risk of the indicated lymphomas. Statistically significant findings depict consistently higher risks for CTCL, NHL, and other lymphomas across all analyses. The boxes correspond to the HR, while error bars represent the 95% CI. More details are shown in the supplement full dataset for this figure. Confidence intervals are shown at the 95% level for interpretability. However, statistical significance was assessed using a Bonferroni-adjusted threshold of α = 0.0071, and readers are advised to interpret confidence intervals in conjunction with adjusted *p*-values.

Since the risk of lymphoma, particularly CTCL, may be confounded by misdiagnosing CTCL as AD, the next step was to evaluate lymphoma risk in non-dermatological T2IDs. In the primary analysis, encompassing over 14.4 million cases and controls, CTCL risk was slightly elevated in a combined group of non-dermatological T2ID cases (HR: 1.38, CI: 1.27–1.50, *p* < 0.0001). Similarly, in T2ID cases, elevated risks were noted for CTCL without Sézary disease (HR: 1.41, CI: 1.30–1.54, *p* < 0.0001), T/NK cell lymphoma (HR: 1.46, CI: 1.37–1.56, *p* < 0.0001), NHL (HR: 1.47, CI: 1.43–1.50, *p* < 0.0001), myeloproliferative neoplasms (HR: 1.66, 95% CI: 1.63–1.69, *p* < 0.0001), and multiple myeloma (HR: 1.48, 95% CI: 1.43–1.53, *p* < 0.0001). No risk difference was observed for Sézary disease. These results remained consistent across both sensitivity analyses (Figure 3).

### Compared to other systemic treatments, dupilumab exposure in non-dermatological T2IDs reduces the risk of non-Hodgkin lymphoma

**Figure 4:**
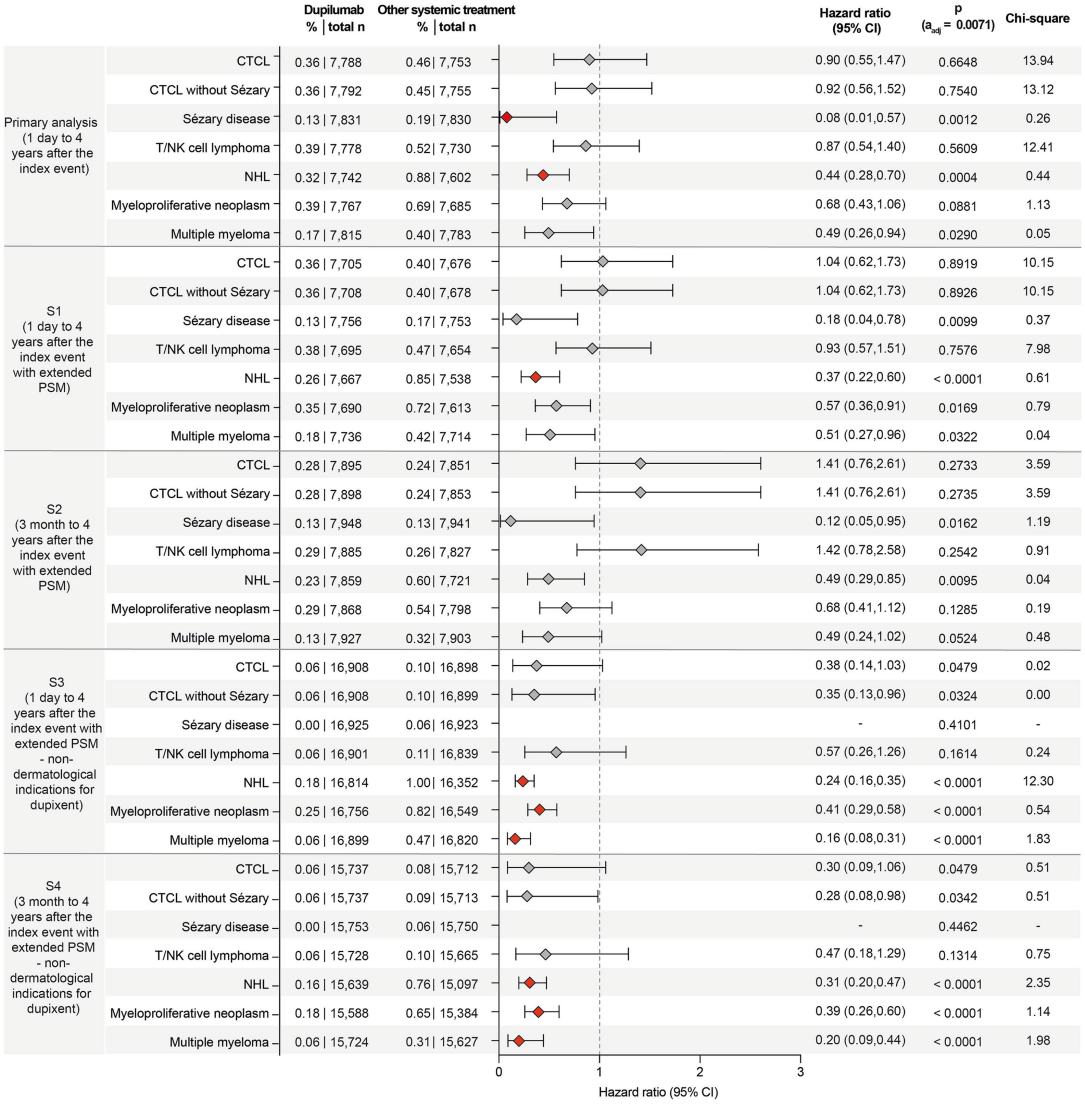
Forest plot of lymphoma risks in patients with AD or T2IDs (and the corresponding controls) treated with either dupilumab or alternative systemic drugs. The forest plots display the hazard ratios (HR) with 95% confidence intervals (CI) for the risk of the indicated lymphomas. Dupilumab treatment does not increase lymphoma risk but is rather associated with reductions in lymphoma risks, e.g., non-Hodgkin lymphoma. The boxes correspond to the HR, while error bars represent the 95% CI. More details are shown in the supplement full dataset for this figure. Confidence intervals are shown at the 95% level for interpretability. However, statistical significance was assessed using a Bonferroni-adjusted threshold of α = 0.0071, and readers are advised to interpret confidence intervals in conjunction with adjusted *p*-values.

Next, the potential impact of dupilumab treatment on lymphoma risk was assessed. In the primary analysis among AD patients, the risk of CTCL, CTCL without Sézary, or Sézary disease was lower in the dupilumab group than in those receiving other systemic treatments, but these differences were not statistically significant. However, the risk of Sézary disease was significantly reduced by over 90% in the dupilumab group (HR: 0.08, 95% CI: 0.01–0.57, *p* = 0.0012). The risk of NHL was also significantly lower in the dupilumab group compared to the control group (HR: 0.44, 95% CI: 0.28–0.70, *p* = 0.0004). For T/NK cell lymphoma and multiple myeloma, no statistically significant differences were observed between the groups (Figure 4). The risk reduction for NHL persisted across all sensitivity analyses, except for sensitivity analysis S2, and in a repetition of the primary analysis in March 2025 (HR: 0.414, CI: 0.225–0.7064, *p* = 0.0036). The reduced risk for Sézary disease observed in the primary analysis only persisted in one of the four sensitivity analyses. These findings demonstrate that dupilumab does not increase the risk of lymphoma but rather tends to reduce the risk of NHL in AD and non-dermatological T2IDs.

Taking a broader view, sensitivity analyses S3 and S4 specifically examined non-dermatological T2IDs where dupilumab is licensed, allowing for a separate assessment of lymphoma risk. If these conditions are considered separately, the potential impact of dupilumab on reducing lymphoma risk is even more pronounced. Specifically, S3 and S4 consistently demonstrated significant reductions in certain lymphoma risks. The risk of NHL was significantly lower in the dupilumab-treated group in both analyses (S3: HR: 0.24, CI: 0.16–0.35, *p* < 0.0001; S4: HR: 0.31, CI: 0.20–0.47, *p* < 0.0001). Similarly, the risk of myeloproliferative neoplasm was significantly reduced in both S3 (HR: 0.41, CI: 0.29–0.58, *p* < 0.0001) and S4 (HR: 0.39, CI: 0.26–0.60, *p* < 0.0001). The risk of multiple myeloma was also significantly lower in both S3 (HR 0.16, CI 0.08–0.31, *p* < 0.0001) and S4 (HR 0.20, CI 0.09–0.44, *p* < 0.0001). For other lymphomas, such as Sézary disease, no cases were observed in the dupilumab-treated group in either analysis. By contrast, the risk reductions for T/NK cell lymphoma and CTCL (with or without Sézary disease) were not statistically significant across both sensitivity analyses (Figure 4).

## Discussion

Foremost, the observed lymphoma rates do not suggest an increased risk associated with dupilumab use. Across multiple analyses, we found no statistically significant evidence of increased lymphoma risk associated with dupilumab use. Of note, confidence intervals include the possibility of both increased and decreased risks. By contrast, for some lymphomas, such as NHL, lower risk estimates were observed among dupilumab-treated patients. These data are in line with a recent report on no increased CTCL risk in AD patients when contrasting AD patients treated with dupilumab compared to other systemic treatment drugs indicated for moderate-to-severe AD (21).

This notion seemingly contrasts with two previous reports on this topic: a retrospective cross-sectional study examined the association between dupilumab treatment, its duration, and the onset of MF in AD patients. The study included 25 patients diagnosed with MF while treated with dupilumab, identified through institutional data and literature review (19). A recent study also investigated the risk of CTCL in AD patients treated with dupilumab (20). The analysis found an increased risk of CTCL in dupilumab-treated patients as compared to non-dupilumab-exposed AD patients. No increased risk was observed for other cutaneous or systemic lymphoid malignancies. The study sparked great interest but also triggered a controversial debate. Concerns focused on selection bias, arguing that comparing dupilumab-treated AD patients, likely patients with moderate-to-severe AD, to non-systemically treated patients could confound the results, as severe AD itself is linked to higher CTCL risk. Concerns were also raised relating to the accuracy of diagnosis, as some CTCL cases could have been misdiagnosed AD, unmasked by dupilumab, rather than genuine adverse events caused by the drug (26–30).

Our study systematically addressed the risk of lymphoma in AD, revealing elevated lymphoma risks in both AD and non-dermatological T2ID patients. These findings align with earlier reports linking AD with an increased lymphoma risk. However, previous studies often did not control for confounding factors, which could have influenced the results (7). Since then, studies have indicated that severe AD is associated with a higher likelihood of developing lymphoma, as demonstrated by a meta-analysis (9, 10). The data presented in this study also extended the so far limited insights into lymphoma risk in non-dermatological T2IDs. While previous research indicated a reduced risk of NHL in asthma patients (12) and identified COPD as a risk factor for MF (13), no prior data had been published on lymphoma risk in non-dermatological T2IDs. The finding presented in this study fill this gap, showing that lymphoma risk is increased in patients with non-dermatological T2IDs. Despite these findings, the overall lymphoma risk in AD and other T2ID patients remains low and does not warrant routine screening for lymphoma in these populations in the absence of symptoms. However, in cases where lymphoma is suspected, precise follow-up is crucial.

This study demonstrates advantages of retrospective cohort studies compared to randomized controlled clinical trials (RCT), which are the gold standard for evaluating the efficacy and safety of both pharmacological and non-pharmacological interventions. However, RCTs are not feasible for all research questions, for example, detection of rare (but clinically relevant) long-term outcomes. Furthermore, in this particular context, it may be ethically challenging to conduct a study comparing the risk of lymphomas between drugs that are otherwise fundamentally different in safety and efficacy (31). Despite these advantages of retrospective cohort studies, we must acknowledge several limitations of this study design in general and for our study in particular: First, it is based on a retrospective analysis of real-world data, which is inherently subject to confounding by unmeasured factors. As a result, observed risk differences are not causal. Additionally, real-world data are vulnerable to errors in ICD-10 code registration and inaccuracies in other variables. This is particularly relevant given the absence of histologic or molecular biological confirmation, leaving diagnoses of skin diseases and neoplasms unvalidated by additional objective measures. Moreover, healthcare accessibility may obscure socioeconomic differences. As some of our analyses included small sample sizes and low event counts, statistical power is decreased, which increases the likelihood of Type II error and limits the precision of effect estimates. Estimates derived from sparse data are more susceptible to random variation and may be less stable across modeling approaches. Therefore, findings in these groups should be interpreted with caution and viewed as hypothesis-generating rather than conclusive. The variables used in this analysis were selected from structured data fields within the TriNetX platform, which only includes patients with complete information for the specified variables. As a result, no missing data were present in the dataset used for propensity score matching or outcome analyses. While this reduces the need for imputation, it may introduce selection bias depending on the internal data processing pipelines of TriNetX. Furthermore, concerns regarding “overpowering” may arise if a large sample size is used (32, 33). Overpowering, however, mostly relates to prospective study designs where excessive sample sizes may lead to detecting statistically significant but clinically trivial differences. In retrospective analyses, the interpretation of results should focus on clinical significance, ensuring that findings are not only statistically meaningful but also relevant to patient outcomes and clinical decision-making. This is underscored by the growing relevance of real-world data in regulatory agencies’ decision-making (31, 34, 35). Finally, the proportionality assumption for Kaplan–Meier (KM) analysis was not met for all comparisons in this study. However, the chi-square and *p*-values calculated from the Schoenfeld residuals were sensitive to differences in time value transformations used by different software packages. For example, Kaplan–Meier time transformations used by Survival and SAS software can yield different chi-square statistics, despite agreement in hazard ratio estimates. This discrepancy arises from the time shifts between transformed values. Thus, TriNetX advises interpreting these chi-square and *p*-values as qualitative guides to the degree of time variance in hazard ratios, rather than as exact quantitative metrics.

In the interim, our findings support the safety of dupilumab. Specifically, there was no observed association between dupilumab treatment and increased lymphoma risk; in contrast, lower NHL rates were associated with dupilumab use relative to other systemic treatments in non-dermatological T2IDs.

## Data Availability

The original contributions presented in the study are included in the article/Supplementary material, further inquiries can be directed to the corresponding author.
