# Supplementary material for: Dupilumab treatment is not associated with changes in lymphoma risk in atopic dermatitis and other type 2 inflammatory diseases: data from a large-scale retrospective cohort study
Source: Front Med (Lausanne). 2026 Jan 20;12:1702736. doi: 10.3389/fmed.2025.1702736 (PMC12864119; doi:10.3389/fmed.2025.1702736)
Supplement: Supplementary file 1 [file Supplementary_file_1.docx]

**Methods**

## **Study design and database**

A propensity-score-matched retrospective cohort study was conducted (**Figure 1**) using the US Collaborative Network from the federated electronic health record (EHR) database TriNetX, following previously published protocols (1–3). This network was chosen due to its large volume of documented EHRs and the comprehensive covariate data available (4). Researchers at UKSH have access to the TriNetX database through a collaboration with TriNetX. The US Collaborative Network of TriNetX was utilized to identify EHRs from patients diagnosed with AD (or non-dermatological T2IDs) and a matched non-AD (non-dermatological T2ID) control group. The risks for CTCL, CTCL excluding Sézary disease, Sézary disease, T/NK cell lymphoma, NHL, myeloproliferative neoplasms, and multiple myeloma were compared between AD patients and non-AD controls using PSM to enhance comparability. Two sensitivity analyses were performed to assess the robustness of the results. To evaluate the potential lymphoma risk associated with dupilumab, these risks were also compared between AD patients treated with dupilumab, non-dermatology T2IDs patients treated with dupilumab, and corresponding groups receiving systemic treatments other than dupilumab. Here, three sensitivity analyses were performed to assess the robustness of the results. Endpoints were defined using ICD-10 codes before data collection.

## **Ethics statement**

The data reviewed is a secondary analysis of existing data, does not involve intervention or interaction with human subjects, and is de-identified per the de-identification standard defined in Section §164.514(a) of the HIPAA Privacy Rule. The process by which the data is de-identified is attested to through a formal determination by a qualified expert as defined in Section §164.514(b)(1) of the HIPAA Privacy Rule. This formal determination by a qualified expert refreshed on December 2020. Thus, our study did not require Institutional Review Board approval.

## **Study population**

The study was conducted from July to October 2024, with data collected from the US Collaborative Network with natural language processing of TriNetX. This network was selected for its high level of data completeness and depth (4). At the time of analysis, the database provided access to EHRs from over 118 million patients across 67 healthcare organizations (HCOs) in the United States. The study included adult patients aged 18 years or older. AD was defined using the ICD-10-CM code L20. Non-AD controls were identified from EHRs documenting a healthcare encounter for general examination without complaint, suspected, or reported diagnosis (ICD-10-CM:Z00), excluding those with ICD-10-CM code L20. The index event was defined as the time of AD diagnosis for cases, or the healthcare encounter for controls. Non-dermatological T2IDs were defined by presence of ICD-10-CM:J32, J33, J44, J45 or K20.0, excluding those with ICD-10-CM:L20. Corresponding controls were defined by ICD-10-CM:Z00, excluding ICD-10-CM:J32, J33, J44, J45, K20.0 and L20.

To delineate the risk of lymphoma, AD patients and those with non-dermatological indications for dupilumab who were exposed to either dupilumab or other systemic treatments were included in the analysis. Non-dermatological indications for dupilumab included asthma (ICD-10-CM:J45), COPD (ICD-10-CM:J44), eosinophilic esophagitis (ICD-10-CM:K20.0), and chronic sinusitis (ICD-10-CM:J33). Drug exposure in the AD groups was identified by the following RxNorm codes: dupilumab (1876376), mycophenolic acid (7145), mycophenolate mofetil (68149), tralokinumab (2589225), cyclosporine (3008), abrocitinib (2591476), azathioprine (1256), and methotrexate (6851). For the non-dermatological dupilumab indication group, drug exposures were similarly defined by RxNorm codes for dupilumab (1876376), theophylline (10438), roflumilast (1091836), mycophenolic acid (7145), mycophenolate mofetil (68149), tralokinumab (2589225), upadacitinib (2196092), cyclosporine (3008), abrocitinib (2591476), azathioprine (1256), and methotrexate (6851). All treatments (and exclusions) were required to occur within four years following the index event.

## **Covariates**

To minimize bias from potentially confounding variables, PSM was performed by establishing a covariate matrix that included relevant concomitant diagnoses, laboratory findings, and demographic information. Covariates included in the analyses are indicated in **Table 1** and **Supplement Tables 1-2**. Matching was performed 1:1 using propensity-scores. Baseline characteristics were reported before and after matching. Due to the sample size the number of covariates for PSM was limited. For a complete description of the groups, additional parameters were retrieved (depicted in the tables in grew) that were, however, not used for PSM.

## **Outcomes**

Outcomes were defined by the respective ICD-10-CM codes: CTCL (ICD10-CM:C84.0, C84.1, C84.A, or C86.6), CTCL - without Sézary disease (ICD10-CM:C84.0, C84.A, or C86.6), Sézary disease (ICD-10-CM:C84.1), T/NK cell lymphoma (ICD-10-CM:C84, or C86), NHL (ICD-10-CM:C82, C83, C85, or C91.1), multiple myeloma (ICD-10-CM:C90.0), and myeloproliferative neoplasms (ICD-10-CM:C92.1, C92.2, D45, D47.1, or D47.3).

## **Primary, sensitivity and subgroup analyses**

In the primary analysis, which contrasted lymphoma risk between AD patients and non-AD controls, outcomes occurring from one day after the index event up to four years post-index were considered. Propensity score matching (PSM) in the primary analysis was based on age, sex, and self-reported White descent (**Table 1**). In sensitivity analysis S1, additional covariates were included in the PSM (**Table 1**). Sensitivity analysis S2 aimed to exclude outcomes that may have been present but undiagnosed or uncoded at the index event by considering only outcomes occurring three months after the index event through four years post-index. The covariates used for PSM are listed in **Table 1**. In the primary analysis comparing lymphoma risk between non-dermatological T2IDs and non-T2ID controls, outcomes occurring from one day after the index event up to four years post-index were considered. PSM was conducted based on age, sex, and self-reported White descent (**Table 2**). Sensitivity analyses S1 and S2 followed the same methodology as described for AD, and cohorts are described in **Supplement Table 1**. To assess the potential lymphoma risk in patients exposed to dupilumab, the primary analysis considered outcomes occurring from one day to four years after the index event. The covariates used for PSM are listed in **Supplement Table 2**. Sensitivity analysis S1 included additional covariates for PSM (**Supplement Table 2**). Sensitivity analysis S2 focused on outcomes occurring three months to four years post-index, using the same covariates for PSM as S1. In sensitivity analysis S3, only patients with non-dermatological indications for dupilumab were included, with follow-up and PSM covariates applied as in S1. Sensitivity analysis S4, was similar to S3 but excluded any outcomes occurring within the first three months following the index event (**Supplement Table 2**).

## **Statistical analysis**

A propensity-score for each patient was generated by logistic regression analysis (with exposure as the dependent variable) using the Python package scikit-learn. Matching was performed 1:1 using the greedy nearest neighbor approach with a cut-off distance of 0.1 pooled standard deviations of the logit of the propensity-score. Baseline characteristics were re-evaluated and reported after matching, differences were compared by t-test for continuous and z-test for binary or categorical variables. Relative risks and risk difference (RD) were calculated. Survival analysis was performed using the Kaplan–Meier method (KM) in Survival package v3.2–3 in R (R Foundation for Statistical Computing, Vienna, Austria) and validated by comparison with the outputs of SAS version 9.4 (SAS, Cary, NC). The proportionality assumption was tested by the cox.zph() function in R's Survival package. KM-curves were compared using the Log-rank test. A univariate Cox proportional hazards regression was used to express hazard ratios (HR)s with 95%-confidence intervals (CI)s. Outcomes prior to index were excluded. Bonferroni correction was used to counter the bias of multiple testing (α[adjust]=0.0071).

## **Use of artificial intelligence**

ChatGPT-4o (OpenAI LCC, San Francisco, California, USA) was used to assist in extracting data from tables and revising sections of the manuscript. However, all extracted data and revisions were thoroughly reviewed and validated by the authors. The authors take full responsibility for the accuracy, integrity, and final content of the manuscript.

## **References**

1. Kridin K, Ludwig RJ. Isotretinoin and the risk of psychiatric disturbances: A global study shedding new light on a debatable story. J Am Acad Dermatol. 2023 Feb;88(2):388–94.

2. Kridin K, Bieber K, Vorobyev A, Moderegger EL, Hernandez G, Schmidt E, et al. Risk of death, major adverse cardiac events and relapse in patients with bullous pemphigoid treated with systemic or topical corticosteroids. Br J Dermatol. 2024 Sep 18;191(4):539–47.

3. Olbrich H, Kridin K, Hernández G, Zirpel H, Sadik CD, Terheyden P, et al. Increased cardiovascular risks and mortality in prurigo nodularis: a global cohort study. EBioMedicine. 2024 May;103:105123.

4. Palchuk MB, London JW, Perez-Rey D, Drebert ZJ, Winer-Jones JP, Thompson CN, et al. A global federated real-world data and analytics platform for research. JAMIA Open. 2023 Jul;6(2):ooad035.
