# Supplementary material for: Dupilumab treatment is not associated with changes in lymphoma risk in atopic dermatitis and other type 2 inflammatory diseases: data from a large-scale retrospective cohort study
Source: Front Med (Lausanne). 2026 Jan 20;12:1702736. doi: 10.3389/fmed.2025.1702736 (PMC12864119; doi:10.3389/fmed.2025.1702736)
Supplement: Supplementary file 2 [file Supplementary_file_2.docx]

# **Supplement Tables**

| **Characteristic** | **Definition** | **Atopic dermatitis** | **Non-atopic dermatitis** | **Std. diff.** | **Atopic dermatitis** | **Non-atopic dermatitis** | **Std. diff.** |
| --- | --- | --- | --- | --- | --- | --- | --- |
|  |  | **(Cases)** | **(Controls)** |  | **(Cases)** | **(Controls)** |  |
| **Primary analysis** |  |  |  |  |  |  |  |
| Number of participants | - | 423.306 | 18,025,971 | - | 400.754 | 400.754 | - |
| Follow-up (days) median (interquartile range) | - | 1,056 (1,121) | 1,231 (1,091) | - | 1,056 (1,121) | 1,141 (1,142) | - |
| Age at Index /years, SD) | - | 45.2 ± 18.7 | 35.3 ± 25.1 | 0.4478 | 45.2 ± 18.7 | 45.2 ± 18.7 | < 0.0001 |
| Female (%) | - | 60,694 | 51,734 | 0.1813 | 60,694 | 60,694 | < 0.0001 |
| White (%) | - | 54.11 | 58.87 | 0.0961 | 54.11 | 54.11 | < 0.0001 |
| **Sensitivity analyses S1 and S2** |  |  |  |  |  |  |  |
| Number of participants | - | 423.306 | 18,025,971 | - | 400.754 | 400.754 | - |
| Follow-up (days) median (interquartile range) | - | 1,056 (1,121) | 1,231 (1,091) | - | 1,056 (1,121) | 1,113 (1,113) | - |
| Age at Index /years, SD) | - | 45.2 ± 18.7 | 35.3 ± 25.1 | 0.4478 | 45.2 ± 18.7 | 45.2 ± 18.7 | 0.0005 |
| Female (%) | - | 60.69 | 51.73 | 0.1813 | 60.69 | 60.69 | 0.0002 |
| Male (%) |  | 34.32 | 43.44 | 0.1879 | 34.32 | 33.16 | 0.0246 |
| White (%) | - | 54.11 | 58.87 | 0.0961 | 54.11 | 54.18 | 0.0013 |
| Black or African American (%) | - | 16.91 | 14.46 | 0.0673 | 16.91 | 17.76 | 0.0224 |
| Hispanic or Latino (%) | - | 7.44 | 10.83 | 0.1176 | 7.44 | 8.00 | 0.0208 |
| Problems related to housing and economic circumstances (%) | ICD10:Z59 | 1.86 | 0.64 | 0.1097 | 1.86 | 1.46 | 0.0313 |
| Problems related to employment and unemployment (%) | ICD10:Z56 | 0.861 | 0.229 | 0.0859 | 0.861 | 0.515 | 0.0419 |
| Personal history of nicotine dependence (%) | ICD10:Z87.891 | 8.31 | 3.72 | 0.1936 | 8.31 | 7.39 | 0.0340 |
| Nicotine dependence (%) | ICD10:F17 | 9.31 | 4.09 | 0.2101 | 9.31 | 9.37 | 0.0019 |
| Alcohol related disorder (%) | ICD10:F10 | 3.58 | 1.57 | 0.1272 | 3.58 | 3.01 | 0.0321 |
| Chronic kidney disease (CKD) (%) | ICD10:N18 | 4.89 | 2.59 | 0.1215 | 4.89 | 4.44 | 0.0214 |
| Chronic lower respiratory diseases (%) | ICD10:J40-J4A | 20.22 | 7.43 | 0.3772 | 20.22 | 13.20 | 0.1892 |
| Body mass index (kg/m2) | TNX:9083 | 29.2 ± 7.41 | 28.1 ± 7.7 | 0.1457 | 29.2 ± 7.41 | 29.5 ± 7.52 | 0.0441 |
| Glucocorticosteroids (%) | VA:HS051 | 16.91 | 14.464 | 0.0673 | 16.91 | 17.758 | 0.0224 |

# **Supplement Table 1**. Characteristics of cohorts investigating the risks of the indicated lymphomas between atopic dermatitis (AD, cases) and non-AD controls. Characteristics in light gray were not separately included for propensity-score-matching. Data were retrieved from the US Collaborative Network of TriNetX on July 10^th^, 2024. ***Abbreviations****: SD: standard deviation, std. diff.: standardized difference*.

|  |  | **Before matching** | | | **After matching** | | |
| --- | --- | --- | --- | --- | --- | --- | --- |
| **Characteristic** | **Definition** | **Non-dermatological T2IDs** | **Non-T2IDs** | **Std. diff.** | **T2IDs** | **Non-T2IDs** | **Std. diff.** |
|  |  | **(Cases)** | **(Controls)** |  | **(Cases)** | **(Controls)** |  |
| **Primary analysis** |  |  |  |  |  |  |  |
| Number of participants | - | 8,256,549 | 14,196,134 | - | 7,254,618 | 7,254,618 | - |
| Follow-up (days) median (interquartile range) | - | 1,067 (1,233) | 1152 (1,152) | - | 1074 (1.232) | 1104 (1,166) | - |
| Age at Index /years, SD) | - | 49.9 ± 18.4 | 31.8 ± 25 | 0.8218 | 48.3 ± 18.2 | 48.5 ± 18.5 | 0.0129 |
| Female (%) | - | 57.80 | 50.20 | 0.1529 | 57.26 | 55.67 | 0.0320 |
| White (%) | - | 66.19 | 57.08 | 0.1883 | 65.12 | 63.48 | 0.0342 |
| **Sensitivity analyses S1 and S2** |  |  |  |  |  |  |  |
| Number of participants | - | 8,401,032 | 14,609,719 | - | 7,325,976 | 7,325,976 | - |
| Follow-up (days) median (interquartile range) | - | 1,064 (1,236) | 1,155 (1,132) | - | 1,076 (1,236) | 1,106 (1,164) | - |
| Age at Index /years, SD) | - | 49.9 ± 18.4 | 32.1 ± 25 | 0.815 | 48.3 ± 18.1 | 49.1 ± 18.8 | 0.0451 |
| Female (%) | - | 57.83 | 50.27 | 0.152 | 56.99 | 56.45 | 0.0108 |
| Male (%) |  | 38.81 | 44.69 | 0.119 | 39.56 | 37.77 | 0.0368 |
| White (%) | - | 66.20 | 56.85 | 0.193 | 65.00 | 63.73 | 0.0265 |
| Black or African American (%) | - | 13.66 | 14.09 | 0.013 | 14.15 | 10.91 | 0.098 |
| Hispanic or Latino (%) | - | 6.91 | 12.63 | 0.193 | 7.15 | 8.32 | 0.0438 |
| Problems related to housing and economic circumstances (%) | ICD10:Z59 | 1.09 | 0.47 | 0.070 | 1.00 | 0.71 | 0.0316 |
| Problems related to employment and unemployment (%) | ICD10:Z56 | 0.28 | 0.13 | 0.034 | 0.26 | 0.20 | 0.0123 |
| Personal history of nicotine dependence (%) | ICD10:Z87.891 | 5.65 | 2.45 | 0.163 | 5.12 | 4.19 | 0.0441 |
| Nicotine dependence (%) | ICD10:F17 | 8.80 | 2.67 | 0.266 | 7.22 | 4.90 | 0.0975 |
| Alcohol related disorder (%) | ICD10:F10 | 2.60 | 1.13 | 0.109 | 2.42 | 1.84 | 0.0407 |
| Chronic kidney disease (CKD) (%) | ICD10:N18 | 3.95 | 1.68 | 0.137 | 3.55 | 2.89 | 0.0377 |
| Chronic lower respiratory diseases (%) | ICD10:J40-J4A | 6.49 | 2.25 | 0.209 | 6.27 | 1.46 | 0.2517 |
| Body mass index (kg/m2) | TNX:9083 | 29.9 ± 7.88 | 27.5 ± 7.54 | 0.320 | 30 ± 7.93 | 28.9 ± 6.87 | 0.1523 |
| Glucocorticosteroids (%) | VA:HS051 | 25.084 | 12.841 | 0.316 | 24.362 | 16.568 | 0.1941 |

**Supplement Table 2**. Characteristics of cohorts investigating the risks of indicated lymphomas between non-dermatological T2IDs and non-T2ID controls. Characteristics in light gray were not separately included for propensity-score-matching. Data were retrieved from the US Collaborative Network of TriNetX on October 3^rd^, 2024. *Abbreviations: SD: standard deviation, std. diff.: standardized difference*.
